# Potential roles of gut microbial tryptophan metabolites in the complex pathogenesis of acne vulgaris

**DOI:** 10.3389/fmicb.2022.942027

**Published:** 2022-07-27

**Authors:** Yukun Huang, Lu Liu, Zhenyu Hao, Lingna Chen, Qian Yang, Xia Xiong, Yongqiong Deng

**Affiliations:** ^1^Department of Dermatology and STD, The Affiliated Hospital of Southwest Medical University, Luzhou, China; ^2^School of Nursing, Chengdu Medical College, Chengdu, China

**Keywords:** tryptophan, gut microbiota (GM), metabolite, acne vulgaris, aryl hydrocarbon receptors

## Abstract

Acne vulgaris is a chronic inflammatory skin disease in which the influence of gut microbiota has been implicated but without clarification of mechanisms. Gut microbiota may exert such an influence via metabolites, particularly those of tryptophan. End metabolites of tryptophan activate receptors, including aryl hydrocarbon, G protein-coupled, and pregnane X receptors to stabilize the immune microenvironment and intestinal mucosal homeostasis. Any impact on the pathogenesis of acne vulgaris remains unclear. The current review collates recent advances concerning potential roles of tryptophan metabolism in mediating skin inflammation, follicular sebaceous gland function and intestinal permeability, all of which influence the pathogenesis of acne vulgaris. The aim was to improve understanding of the pathogenesis of acne vulgaris and to expose therapeutic opportunities.

## Introduction

Acne vulgaris is a highly prevalent cutaneous inflammatory disorder that primarily affects the face, chest, and back. Approximately 85% of sufferers are aged 12–24 years with 50% being 20–29 years (Eichenfield et al., 2021). Years of continuous research have established the complexity of acne vulgaris pathogenesis. Dysfunctions of the hair follicle sebaceous glands (Moradi Tuchayi et al., 2015), altered sebum fatty acid composition (Zouboulis et al., 2014), hormonal disorder of the microenvironment (Zouboulis, 2020), neuropeptide interaction (Ganceviciene et al., 2009), abnormal follicular epithelial differentiation, excessive follicle keratinization (Cong et al., 2019), induced inflammation, innate immunity, and adaptive immune system dysfunction (Tanghetti, 2013; Zouboulis et al., 2014) are all thought to be involved. However, the exact pathogenesis of the disease remains unknown. Numerous studies have shown that gut microbiota (GM) affect immune regulation of distant organs, such as lung (Ma et al., 2021), brain (Mayer et al., 2022), and skin (De Pessemier et al., 2021). Advances in new-generation sequencing technologies over the past decade have allowed unprecedented insights into the human microbiome (Zhernakova et al., 2016). We have previously conducted an analysis of the GM in acne vulgaris patients and found significant deviations from that of the healthy, including decreased abundance of *Lactobacillus* and *Bacillus* (Deng et al., 2018). GM analysis in moderate and severe acne vulgaris patients found that the abundance of various probiotics, such as *Bifidobacterium* and *Lactobacillus*, decreased (Yan et al., 2018). These observations strongly imply an interaction between GM and the onset of acne vulgaris but no specific mechanism has been clarified.

Many effects of GM are known to be mediated by metabolites derived from environmental transformation or generated by microorganisms. Dysregulation of aminoadipic acid metabolism has been suggested to be associated with increased risk of acne vulgaris (Lin et al., 2019) and the essential amino acid, tryptophan (Trp), is an aminoadipic acid metabolite. Tryptophan has essential physiological roles and has also been implicated in the development of other diseases, such as metabolic syndrome, obesity and depression (Bosi et al., 2020). The current brief review summarizes potential roles of microbial Trp metabolites in regulating local skin inflammation, hair follicle sebaceous gland dysfunction and gut permeability. The aim was to improve the comprehension of acne vulgaris development and expose therapeutic opportunities.

## Gut microbiota and acne vulgaris

The human microbiome comprises a wide range of microorganisms that inhabit the body and protect it against external intrusions through an impact on immunity. Studies of microbiome composition have been hindered by its complex nature and the difficulties in culturing some of the components. However, recent advances in research technologies, such as 16S rRNA sequencing, metagenomics, metabolomics and metaproteomics, have enabled microbiome-associated research (Jangi et al., 2016; Zhernakova et al., 2016). As a result, changes in GM composition and diversity have been linked to the gut immune environment and barrier function and been found to affect the inflammatory response of distant organs (Gilbert et al., 2018). Common factors contributing to GM changes are psychosocial stress, social relationships, health, alcohol intake, tobacco use and diet (Dowd and Renson, 2018). A Western diet, enriched in carbohydrates, saturated fats and salt, has been linked to acne development (Baldwin and Tan, 2021). GM analyses have identified *Bacteroidetes* as a phylum positively associated with fat but negatively associated with fiber and *Firmicutes* as having the opposite association (Jangi et al., 2016). Westernization of the diet promotes GM dysbiosis with attendant changes in gut permeability and barrier function and leads to abnormal activation of immune cells which may eventually contribute to the development of chronic diseases (Zinocker and Lindseth, 2018). Reduced levels of *Lactobacillus* may mediate the hypertensive effects of high-salt diets in humans and experimental animals (Na et al., 2021). Additives, such as artificial sweeteners, are associated with intestinal permeability, GM changes and inflammation, promoting an increase in pathogenic bacteria (enterobacteria) and a reduction in beneficial bacteria (*lactobacilli* and *bifidobacterial*; Schiffman and Nagle, 2019). Recent studies have shown that patients with acne vulgaris exhibited lower *Firmicute*s and increased *Bacteroides* with potentially beneficial taxa, such as *Lactobacillus, Bifidobacterium* and *Bacillus* being depleted (Deng et al., 2018; Yan et al., 2018).

GM are known to participate in the metabolism and absorption of food through the human digestive system and also to generate GM-host co-metabolites, such as short-chain fatty acids (SCFAs), Trp metabolites, lipids and bile acids. Such metabolites are necessary for immune homeostasis and influence the host’s susceptibility to many immune-mediated disorders. For example, SCFAs, such as butyrate, acetate, and propionate, bind “metabolite-sensing” G protein-coupled receptors (GPCRs), including GPCR41, GPCR43, and GPCR109A, to inhibit histone deacetylases and promote a tolerogenic, anti-inflammatory cell phenotype (Cummings and Macfarlane, 1997; McKenzie et al., 2017). Bile acids bind to G protein-coupled bile acid receptor 1 to exert anti-inflammatory effects in the gut, promoting the anti-inflammatory M2 macrophage phenotype and reducing the pro-inflammatory M1 macrophage phenotype. Secondary bile acids modulate the Treg/Th17 ratio to regulate the adaptive immune system (Ridlon et al., 2014; Ramirez-Perez et al., 2017; Fiorucci et al., 2018). Recent research on the relationship between acne vulgaris and GM (Bowe and Logan, 2011; Yan et al., 2018; Lin et al., 2019; Szegedi et al., 2019; Huang et al., 2021) has largely been limited to correlation analysis without addressing causal relationships. A more complete understanding of the relationship between GM-associated metabolites and acne vulgaris could indicate new directions for diagnosis and management of the disorder.

## Bacterial tryptophan metabolism in the gut

Trp is an essential amino acid abundant in high-protein foods, such as eggs, meat, fish, cheese, and nuts. The small intestine digests and absorbs most ingested protein, although some proteins and amino acids may arrive at the colon depending on the intake (Evenepoel et al., 1999). Trp is not only metabolized but also synthesized by GM (Paley, 2019). Trp released by the small intestine circulates in its free form or binds to albumin in the peripheral bloodstream. Trp metabolism in the gastrointestinal tract is divided into three main pathways (Table 1): (i) Gut bacteria convert Trp into indoles, indole derivatives and tryptamine which are ligands of aryl hydrocarbon receptors (AhR); (Zelante et al., 2013); (ii) the kynurenine pathway, including indoleamine 2,3-dioxygenase 1 in immune and epithelial cells (Clarke et al., 2013) and (iii) serotonin production in intestinal chromaffin cells through tryptophan hydroxylase 1 (Yano et al., 2015).

**TABLE 1:** Summary of tryptophan metabolic pathways in the human body.

Pathways	Enzymes	Key metabolites	Bacteria/Host	References
Kynurenine pathway (∼95%)	TDO/IDO	Kyn, KA, QA	Both	Savitz, 2020
Indole pathway (∼5%)	ArAT	Indole, IAId, ILA, IPA, IAA, IA, tryptamine	Bacteria	Taleb, 2019
Serotonin pathway (∼1–2%)	TPH1/2, AAAD, MAO	5-HT, Melatonin	Host	O’Mahony et al., 2015

AAAD, aromatic amino acid decarboxylase; ArAT, aromatic amino acid aminotransferase; 5-HT, 5-hydroxytryptamine; IA, Indole-3-acrylic acid; IAA, indoleacetic acid; IAld, indole-3-aldehyde; IDO, indoleamine 2,3-dioxygenase; ILA, indole-3-lactic acid; IPA, indole-3-propionic acid; KA, Kynurenic acid; Kyn, Kynurenine; MAO, monoamine oxidase; QA, Quinolinic acid; TDO, tryptophan 2,3-dioxygenase; TPH, tryptophan hydroxylase.

GM play a vital role in the digestion and absorption of amino acids and commensal bacteria in the colon convert Trp to indole and its derivatives, such as IAId (indole-3-aldehyde), ILA (indole-3-lactic acid), IPA (indole-3-propionic acid), IAA (indoleacetic acid), IA (Indole-3-acrylic acid) and tryptamine, which have roles in maintaining intestinal immune homeostasis and barrier function (Jennis et al., 2018). *Lactobacillus murinus* and *Lactobacillus reuteri* have been reported to convert Trp to IAld and ILA via aromatic amino acid aminotransferase and indole lactic acid dehydrogenase (Cervantes-Barragan et al., 2017; Wilck et al., 2017). Moreover, several *Bacteroides*, in addition to *Clostridium*, have been reported to generate indole, IPA, IA, ILA, IAA, and tryptamine (Aragozzini et al., 1979; Russell et al., 2013; Devlin et al., 2016; Dodd et al., 2017; Paley, 2020; Paley, 2021).

## Tryptophan metabolites and their receptors

Trp has recently been implicated in the crosstalk between GM and the host in healthy and diseased states (Agus et al., 2018; Roager and Licht, 2018). Impaired Trp metabolism may influence many diseases, such as metabolic syndrome, obesity, neuropsychiatric disorders and inflammatory bowel diseases (Agus et al., 2018). We have also found disordered metabolites of aminoadipic acids, such as alanine, histidine, leucine, methionine, serine, and Trp, in acne vulgaris patients (Deng et al., 2018). Trp metabolism end-products may activate the immune system through binding to AhR, GPCRs and the pregnane X receptor (PXR; Agus et al., 2018; Roager and Licht, 2018). AhR is known to be a ligand-dependent transcription factor (Abel and Haarmann-Stemmann, 2010; Gargaro et al., 2021) with numerous roles in skin physiology and disease (Szelest et al., 2021). GPCRs are highly expressed by epithelial cells and specific subsets of immune cells and are thought to recognize GM-derived SCFAs and tryptamine in addition to Trp-derived kynurenic acid (KYNA), the chemokine, CXCL17, and phospholipid-derived lysophosphatidic acid in order to regulate intestinal homeostasis (Maravillas-Montero et al., 2015; Bhattarai et al., 2018; Gao et al., 2018; Kaya et al., 2020). Moreover, AhR and PXR are both involved in intestinal epithelial barrier fortification (Agus et al., 2018). Therefore, the remainder of this review focuses on potential roles of gut microbial Trp metabolites in the complex pathogenesis of acne vulgaris through the above receptors.

## The beneficial role of microbial tryptophan in acne vulgaris-associated disorders

Acne vulgaris sometimes accompanies metabolic syndrome, obesity, and neuropsychiatric disorders. A cross-sectional study of 89 women with acne found that 36% had metabolic syndrome (Gayen et al., 2021) and higher prevalence of insulin resistance and metabolic syndrome occurred in acne sufferers than in controls (Nagpal et al., 2016). Additional previous studies have shown that psychosomatic problems, such as anxiety and depression are linked to the onset of acne (Samuels et al., 2020; Sood et al., 2020). A cross-sectional survey in New Zealand found that students with acne experience higher rates of anxiety (9%), depressive symptoms (24%), suicidal thoughts (34%) and suicide attempts (13%) than those without (Halvorsen et al., 2011). The association between these diseases and acne vulgaris warrants further understanding of the role of Trp.

### Metabolic syndrome and obesity

Metabolic syndrome comprises numerous cardiovascular risk factors, including insulin resistance, obesity and hypertension (Huang, 2009). Fecal samples from patients with metabolic syndrome displayed decreased levels of Trp metabolites (Liu X. et al., 2020) and deficiencies in ligands of AhR have been observed in animal models of metabolic syndrome. Compensatory administration of *Lactobacillus* or an AhR agonist reduced glucose dysmetabolism and liver steatosis in animal models with high Trp metabolizing capabilities (Natividad et al., 2018). Intestinal barrier defenses and MyD88 are impaired in diabetes and ICAM and FMO3 expression induced while AhR ligands reverse diabetes-induced intestinal barrier damage, insulin insensitivity, FMO3/ICAM expression and systemic inflammation (Liu W. C. et al., 2020). Indole has been shown to stimulate enteroendocrine L-cells to produce glucagon-like peptide-1, stimulating insulin secretion by pancreatic β-cells. Significant upregulation of AHR mRNA in peripheral blood monocytes of type 2 diabetics has been found and increased ANR transcription correlated with the elevated plasma levels of IL-22 and IL-17 in peripheral blood mononuclear cells from both metabolically healthy obese and type 2 diabetic subjects (Zhao et al., 2020). Thus, Trp metabolites may influence metabolic and pro-inflammatory states in metabolic syndrome and obesity through interactions with AhR.

### Neuropsychiatric disorders

The GM may affect the brain and neuropsychiatric disorders via regulation of circulating Trp which may cross the highly selective blood-brain barrier and affect neurotransmitter metabolism (Agus et al., 2018). Depression is a neuropsychiatric disorder strongly associated with acne vulgaris (Molla et al., 2021) and stems from decreased availability of Trp and serotonin in the brain (Agus et al., 2018). Decreased KYNA in the periphery and the cerebro-spinal fluid of patients after suicide attempts have also been found (Wiedlocha et al., 2021).

Autism spectrum disorder, a severe neurodevelopmental condition, has also been linked to altered GM and Trp metabolism (Kaluzna-Czaplinska et al., 2019). Polymorphisms in the gene encoding the AhR nuclear translocator have been associated with the severity of autism spectrum disorder (Fujisawa et al., 2016). Nevertheless, no links have been confirmed between GM species and specific mechanisms. We could still speculate that the gut-brain-skin axis has a vital role in some acne-associated psychiatric disorders whose pathogenesis is partly due to the changes in altered GM and Trp metabolism.

## Probiotics may treat acne vulgaris by modulating tryptophan metabolism

Probiotics are live microorganisms that exert beneficial effects on the host when adequately ingested. Promising results have emerged from the application of probiotics for skin diseases, such as acne vulgaris, atopic dermatitis and allergies (Goodarzi et al., 2020; Yu et al., 2020). Twelve week oral administration of *Lactobacillus bulgaricus* and *Streptococcus thermophilus* produced a 33.2 percent reduction in total acne lesion counts in 56 adults (Kim et al., 2010) and 12 weeks of oral administration of a mixed probiotic preparation of *Lactobacillus acidophilus*, *Lactobacillus delbrueckii* subspecies *bulgaricus* and *Bifidobacterium biftdum* reduced acne lesion counts by 67 percent in 45 adults (Jung et al., 2013). A randomized blind-controlled trial in which 20 adults with acne were given a supplement containing *Lactobacillus rhamnosus* for 12 weeks produced “improvement/significant improvement” in acne scores which were not seen in the placebo group. Probiotic intervention also decreased IGF-1 and increased FoxO1 expression (Fabbrocini et al., 2016). The PI3K/Akt/FoxO1/mTORC1 of the Mammalian Target of Rapamycin (mTOR) C1 pathway induced by IGF-1 is known to be involved in the pathogenesis of acne (Melnik, 2018; Cong et al., 2019).

Research also confirmed the relationships between GM and Trp metabolism. *Lactobacillus* levels were found to be diminished by chronic stress in parallel with elevated serum kynurenine, effects which were reversed by restoration of gut *Lactobacillus*. Moreover, attenuation of the proinflammatory response and increased serum Trp and KYNA were observed after prolonged *Bifidobacterium infantis* administration in rats (Wiedlocha et al., 2021). Indeed, oral feeding of *Lactobacillus reuteri* to healthy breastfed mice significantly increased IPA and IA (Liu et al., 2022) and administration of *Lactobacillus salivarius* to germ-free rats caused generation of Trp-derived ILA (Xia et al., 2021).

Based on the role of the GM in Trp metabolism, beneficial effects of Trp metabolites in acne vulgaris-associated disorder and clinical probiotic treatment of acne vulgaris, we could speculate that the increased intestinal probiotics, such as *Lactobacillus* and *Bifidobacterium*, may generate Trp-derived end metabolites which inhibit immune inflammation, regulate sebum synthesis, and alleviate intestinal leakage.

## Microbial tryptophan metabolites may regulate innate and adaptive immune responses, and inhibit sebum synthesis in acne vulgaris

**FIGURE 1:**
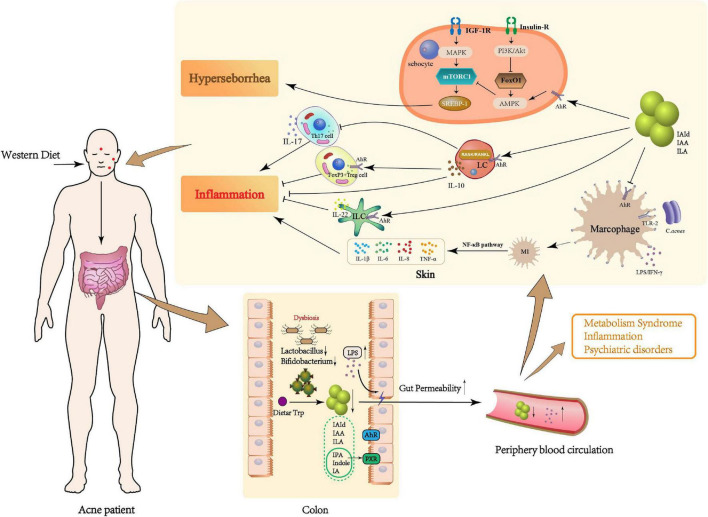
Potential role of microbial Trp metabolites in the pathogenesis of acne vulgaris. Patients with acne vulgaris on a long-term Western diet may develop GM dysbiosis, which could result in a decrease in beneficial bacteria (e.g., *Bifidobacterium, Lactobacillus*), an essential metabolic link in the dietary Trp metabolism, and a reduction in potentially beneficial metabolites such as IAId, IAA, and ILA. In the local skin, Trp metabolites not only inhibit the production of pro-inflammatory factors such as IL-1β, IL-6, IL-8, and TNF-α by Mφ differentiating to M1 type through AhR but also promote the secretion of anti-inflammatory factors such as IL-10 and IL-22 by LC and ILC through AhR, and induce the differentiation of CD4+T cells to FoxP3+Treg and inhibit Th17 cells. On the other hand, Trp metabolites may also reduce sebum synthesis by inhibiting mTORC1, thus alleviating acne vulgaris. The “gut-skin” link may be caused by the disruption of the intestinal barrier caused by GM dysbiosis. Increased LPS in the gut may also enter the local skin through the peripheral circulation and aggravate the occurrence of acne vulgaris.

The pathogenesis of acne vulgaris includes follicular hyperkeratinization, hyperseborrhea, increased *C. acnes* colonization and inflammation (Kurokawa et al., 2009). Indeed, inflammation participates at all stages of acne development, from microcomedo and open/closed comedones to inflammatory comedones and even acne scars (Tanghetti, 2013; Dreno et al., 2015; Cong et al., 2019). The role of adaptive and innate immune systems in regulating inflammation during acne pathogenesis via Toll-like receptors (TLRs) has been established. By this mechanism, inflammasomes are activated, matrix metalloproteinases produced, and antimicrobial peptide activity stimulated (Kistowska et al., 2014; Dreno et al., 2015). The adaptive response can also induce inflammation. Specific anti-*C. acnes* activate complement and recruit neutrophils (Dreno et al., 2015). CD4 + T cells were also found in patches of acne where they exacerbate inflammation (Agak et al., 2014; Kelhälä et al., 2014; Kistowska et al., 2014; Eliasse et al., 2021). An exploration of roles and mechanisms of GM Trp metabolism in mediating innate immune inflammation and sebaceous gland function during acne vulgaris pathology follows (Figure 1).

### Microbial tryptophan metabolites may suppress inflammation in acne vulgaris by regulating the innate immune response

Skin is the body’s first line of defense against environmental threats and pathogen invasion. It consists of a complex and diverse network of epithelial, immune and neuronal cells that integrate signals (Abreu and Kim, 2021). Skin immunity is coordinated by innate and adaptive immune cells with the former, including macrophages (Mφ), basophils, mast cells, and innate lymphoid cells (ILCs), responding rapidly to non-specific stimuli. The inflammatory response mediated by innate immune cells is considered an essential pathological link in the development of acne vulgaris (Dreno et al., 2015; Cong et al., 2019).

#### Macrophages

Mφ differentiate into two phenotypes, the inflammatory M1 phenotype activated by the TLR ligands, interferon-gamma or lipopolysaccharide (LPS) and the alternative anti-inflammatory M2 phenotype (Mosser and Edwards, 2008). Colonization by *C. acnes* would activate mononuclear Mφ via surface TLR2/4, recruit MyD88 signaling molecules and activate the NF-κB inflammatory signaling pathway to release inflammatory factors, IL-6, IL-8, IL-1β, and TNF-α (Cong et al., 2019). The Trp metabolite receptor, AhR, influences Mφ polarization and the regulation of immune function. LPS-induced endotoxic shock is more profound in AhR-null mice, compared to WT, and Mφ from these mice produce higher levels of proinflammatory cytokines (Kimura et al., 2009). Deletion or inhibition of AhR in Mφ co-cultured with apoptotic cells shifts the IL-10-dominated anti-inflammatory response to a pro-inflammatory state with increased IL-6, IL-12p40, and TNF-α. The end Trp metabolite, indole-3-acetate, reduced LPS- and fatty-acid-stimulated production of pro-inflammatory cytokines in macrophages and inhibited cell migration toward chemokines (Krishnan et al., 2018). Moreover, it is reported that the IAId-stimulation of LPS-activated primary human Mφ decreased IL-6 signaling (Walter et al., 2021). Tryptamine and KYNA also bind GPCRs and influence Mφ polarization to mediate the inflammatory process (Vieira et al., 2016; Husted et al., 2017).

#### Innate lymphoid cells

ILCs mediate innate and adaptive immunity and participate in the pathogenesis of various autoimmune diseases (Gwela et al., 2017; Withers and Hepworth, 2017; Sonnenberg and Hepworth, 2019; Satoh-Takayama et al., 2020). Tissue-resident ILCs maintain mucosal tissue homeostasis when internal physiological responses are disrupted. The Western-style high-calorie diet may induce transcriptional and epigenetic reprogramming of monocytes and enhance myeloid progenitor cell proliferation (Christ et al., 2018). Unsurprisingly, the AhR is also involved in the secretion of IL-22 by ILCs (Martin et al., 2009; Veldhoen et al., 2009; Cibrian et al., 2016). Activation of AhR by IAId induces IL-10 production by Langerhans cells (LCs) and RANK and RANKL expression by LCs and keratinocytes, respectively, leading to NF-kB signaling and IL-10 production.

Moreover, LCs activated by IAId inhibit CD4^+^ T cell proliferation and induce IL-10 secretion (Liu X. et al., 2020). Although distinct differences in innate lymphoid cell expression in skin lesions of acne patients compared with healthy controls have not been confirmed, secretion of anti-inflammatory IL-10 by peripheral blood mononuclear cells is reduced in acne patients and adding exogenous IL-10 to peripheral blood mononuclear cell cultures from acne patients restores its phagocytic activity. These observations suggest that IL-10 modulates pro-inflammatory cytokines and may have utility in treating acne vulgaris (Caillon et al., 2010).

### Microbial tryptophan metabolites may suppress acne vulgaris inflammation by regulating adaptive immune responses

Adaptive immunity is also considered to influence the pathogenesis of acne vulgaris. CD4 + T cells have two main subtypes: pro-inflammatory type IL-17 generating cells and anti-inflammatory FOXP3 + Treg cells (Elias et al., 2008). T lymphocytes have been demonstrated to appear first in early acne lesions (Norris and Cunliffe, 1988; Jeremy et al., 2003) and IL-17-producing cells with increased IL-17A tissue expression have also been found in lesions (Agak et al., 2014; Kistowska et al., 2014). Activation of mast cells to produce IL-17 in CD4 + T cell rich areas has been suggested, even in the subclinical stages of acne (Eliasse et al., 2021), and scattered distribution of IL-17A positive cells was observed in both the papillary dermis and around sebaceous follicles in acne lesions (Kelhälä et al., 2014).

AhR regulates the balance among various T cell populations, proinflammatory or autoimmune Th17 cells, immunosuppressive or tolerogenic Treg and T regulatory type 1 (Tr1) cells, and is affected by the cellular microenvironment and specific ligands. Activation of AhR by tetrachlorodibenzo-p-dioxin promoted FoxP3 gene transactivation *in vitro* and expanded the CD4 + CD25 + FoxP3 + Treg-cell compartment *in vivo*. AhR activation has been reported to increase Tregs to reduce inflammation and ameliorate disease (Quintana et al., 2008; Quintana et al., 2010; Singh et al., 2016; Abron et al., 2018; Al-Ghezi et al., 2019; Alrafas et al., 2019). Epigenetic changes in the FoxP3 locus and expression of additional transcription factors required to induce functional FoxP3 + Treg cells are the result (Gandhi et al., 2010; Singh et al., 2011). AhR activity is synergistic with that of c-Maf and transactivated IL-10 and IL-21 in Tr1 cells (Gandhi et al., 2010; Mascanfroni et al., 2015) and facilitated RORγt-mediated IL-22 transcription in Th17 cells (Veldhoen et al., 2008; Veldhoen et al., 2009). Additionally, GPCR61 was expressed at higher levels in the Th17 cell subset compared to resting CD4 + cells which may indicate a potential role for this receptor in autoimmune diseases (Kozielewicz et al., 2017). Tregs homing reactions mediated by GPCR -ligand interactions may also aid the maintenance of intestinal immune homeostasis (Song et al., 2022).

The above may indicate regulation of the skin’s innate and adaptive immune systems by bacterial Trp metabolites. Microbial Trp metabolites may enter the peripheral circulation through the intestinal mucosa in the area of acne lesions, activate the AhR and GPCR-induced signaling, attenuating Mφ polarization, activation of ILCs and secretion of inflammatory factors by Treg cells. This sequence of events would alleviate the acne-induced inflammation. Deficiency of intestinal probiotics in acne vulgaris patients would lead to decreased Trp metabolites, such as IAId, IAA, and ILA, and weakened activation of AhR and GPCR on the Mφ surface. The Mφ would then be prone to polarization into the M1 phenotype with promotion of the local inflammatory response. Compensatory intestinal probiotics or microbial Trp metabolites would maintain AhR activation and the anti-inflammatory microenvironment of M2 phenotype-Mφ. Alternatively, Trp metabolites may induce ILCs, through AhR and GPCR activation, to produce a protective anti-inflammatory cytokine, IL-10, and regulate the inflammatory environment. Direct relationships between probiotics/Trp metabolites and acne vulgaris require further investigation. Little has been reported regarding the role of microbial Trp metabolites and GPCR-mediated immune inflammation, an area which is worthy of further scrutiny.

### Microbial tryptophan metabolites may inhibit sebum synthesis and promote the resolution of acne vulgaris

The PI3K/Akt/FoxO1/mTORC1 axis of the Mammalian Target of Rapamycin (mTOR) C1 pathway, induced by IGF-1, is known to be an essential signaling pathway for the pathogenesis of acne (Melnik, 2018; Cong et al., 2019). Both improvements in skin lesions and decreased insulin levels are associated with a low-glycemic diet (Smith et al., 2007). By contrast, a high-glycemic diet and excessive dairy product intake may activate insulin/IGF-1 signaling, stimulating mTORC1 and enhancing keratinocyte proliferation, and hormone production, leading to acne vulgaris (Emiroglu et al., 2015). Extensive research has suggested an impact of long-term dietary tendency on alterations in GM, predisposing individuals to insulin resistance and higher serum insulin (Del Prete et al., 2012; Clark et al., 2017). For instance, GM-induced imidazole propionate in type 2 diabetes may impair insulin signaling via p38γMAPK activation, promoting p62 phosphorylation and mTORC1 activation (Koh et al., 2018). Therefore, acne occurrence is not limited to hair follicles and sebaceous glands: GM imbalance also has an impact.

In addition, activation of the AhR signaling pathway appears to inhibit human sebocytes, reducing sebum production (Hu et al., 2016). An *in vitro* study on the sebocyte AhR showed that its stimulation resulted in attenuated expression of lipogenic genes via enhanced SREBP1 proteolysis (Muku et al., 2019). Cross-talk between AhR and TLR-2 was probably responsible for this (Hou et al., 2019). The AhR agonist, peptidoglycan, stimulates TLR-2 on cultured human sebocytes to induce TNF-α and IL-8 secretion, an effect which was inhibited after AhR knockdown and pre-treatment with an AhR antagonist (Hou et al., 2019). Moreover, *C. acnes*–Mφ interactions may depend on sebum composition with pathological and therapeutic relevance for acne (Lovaszi et al., 2017). Overall, these findings indicate roles of microbial Trp metabolites as AhR ligands which affect lipid synthesis and immune cell differentiation, raising the possibility of therapeutic uses for acne vulgaris treatment. Further confirmatory studies need to be conducted.

## Microbial tryptophan metabolites could stabilize the intestinal mucosa, reduce intestinal leakage, and promote acne regression

The intestinal barrier is the first line of defense against hazardous pathogens and dysfunction results in enhanced permeability and translocation of microbial entities, such as LPS, to the systemic circulation, leading to inflammation (Cani et al., 2008). Stokes and Pillsbury discovered a link between the microbiome and skin inflammation as early as the 1930s (Stokes and Pillsbury, 1930). They found hypochlorhydria in a majority of acne patients, a lack of acid which promoted the migration of bacteria from the colon to the small intestine, disrupting GM structure. More recently, hypochlorhydria was identified as a significant risk factor for small intestinal bacterial overgrowth, enhancing intestinal permeability and systemic inflammation (Lombardo et al., 2010; Reddymasu et al., 2010). Thus, altered GM of acne patients may disrupt the intestinal barrier, leading to the entry of endotoxin into the peripheral blood circulation and further pathological changes.

LPS, an abundant gut endotoxin, contributes to impaired intestinal epithelial barrier and immune function (Nicholson et al., 2012). It binds TLR-4 to stimulate an extreme inflammatory reaction (Candelli et al., 2021). We have previously found increased *Bacteroidetes* and *Escherichia coli* and markedly over-expressed LPS biosynthesis pathways in the GM of acne patients (Deng et al., 2018). *Bacteroidetes* and *Escherichia coli* are thought to be the main contributors to LPS biosynthesis (Juhlin and Michaelsson, 1983; Hevia et al., 2014; Deng et al., 2018). Altered GM may contribute to the phenomenon of a “leaky gut.” LPS is a pro-inflammatory molecule produced by gram-negative bacteria which may promote increased intestinal permeability and decreased expression of intestinal tight junction proteins. LPS-binding protein-4 activates TLR-4, allowing LPS to transit from the intestinal lumen into the bloodstream and bind the serum LPS receptor, CD14 (Guo et al., 2013; Guo et al., 2015; Park et al., 2016). Thus, LPS from a “leaky gut” may enter the blood and mimic the TLR activation by *C. acnes* and other bacteria, exacerbating inflammation and promoting cytokine cascades.

Trp metabolites may also be beneficial for intestinal barrier stability. Previous studies have shown that the feces of obese and untreated type II diabetic subjects contain more kynurenic acid, an end product of Trp metabolism (Laurans et al., 2018). A similar pattern has been reported for intestinal inflammatory diseases (Lamas et al., 2016), suggesting a common mechanism of a disrupted intestinal barrier. Reduced AhR activation has been observed in the GM of individuals with metabolic syndrome and intestinal inflammatory disease (Lamas et al., 2016; Natividad et al., 2018). However, indoles can reinforce intestinal epithelial barrier function by increasing the expression of genes involved in preserving epithelial cell structure and function *in vivo* and *in vitro* (Bansal et al., 2010; Shimada et al., 2013). IA may promote intestinal goblet cell differentiation and mucus production through AhR activation, thereby regulating intestinal epithelial barrier function and alleviating inflammatory responses in mice (Wlodarska et al., 2017). In addition, bacterial metabolites of Trp which are PXR ligands, such as IPA, Indole and IA, modulated intestinal barrier function in mice (Venkatesh et al., 2014; Illes et al., 2020). PXR has been reported to be activated by Trp metabolites, such as IPA and indole, strengthening intestinal barrier function (Niu et al., 2022), alleviating colonic inflammation (Huhn et al., 2020) and protecting against LPS-induced muscle inflammation (Du et al., 2021). Homing reactions of Tregs, mediated by GPCR -ligand interactions, have been thought to play a central role in maintaining intestinal immune homeostasis (Song et al., 2022). Thus, GM-induced Trp metabolites bind AhR and PXR to modulate GM composition, activate the immune system and repair the intestinal epithelial barrier, thereby exerting anti-inflammatory, antioxidative or toxic effects in the systemic circulation (Roager and Licht, 2018).

Decreased abundance of beneficial taxa in the gut of acne vulgaris patients may lead to adverse changes in Trp metabolites, reducing intestinal barrier stability and resulting in LPS entering the systemic circulation, followed by skin inflammation. Supplementation with Trp metabolites may be prophylactic for acne morbidity by protecting the gut epithelial barrier. However, mechanisms of Trp metabolism by the intestinal microbiome and its effect on acne pathogenesis remain unclear. More research is needed to provide tangible connections between Trp catabolites and acne vulgaris.

## Discussion

The current elucidation of interactions between the gut microbiome and host may contribute to a better understanding of acne vulgaris and inform further investigation. Changes in the GM, whether in diversity or abundance of specific bacteria, indeed play a vital role in the pathogenesis of acne vulgaris. Decreased probiotics in the intestinal tract of acne vulgaris patients, reduce microbial Trp metabolites which would otherwise alleviate both innate and adaptive immune inflammation, downregulating insulin and IGF-1/mTORC1 signaling, promoting the AhR inhibition of sebum production and enhancing intestinal mucosa stability. Supplementation with probiotics may restore Trp metabolism, ameliorating acne vulgaris through an impact on immunity and inflammation. However, causal relationships between GM and acne vulgaris remain to be established, along with the role of GM-associated Trp metabolism and underlying molecular mechanisms. Only preliminary discussions on correlations between GM metabolism and acne pathogenesis have been reported to date. Experiments involving rat models of acne, fecal transplantation and intestinal organoids are all planned in order to investigate causal relationships between GM metabolites and acne vulgaris. We hope that these findings will provide new insights into the pathogenesis and therapeutic direction of acne vulgaris.

## Author contributions

YH and LL planned the work and wrote the first draft of the manuscript. LC made the figure. ZH assisted with literature review and manuscript revision. YD, QY, and XX seriously revised and edited the manuscript prior to submission. All authors contributed to the article and approved the final submitted version.
